# Stem cell marker CD271 is expressed by vasculogenic mimicry-forming uveal melanoma cells in three-dimensional cultures

**Published:** 2012-03-02

**Authors:** Klara Valyi-Nagy, Bernadett Kormos, Mohamed Ali, Deepak Shukla, Tibor Valyi-Nagy

**Affiliations:** 1 Department of Pathology, University of Illinois at Chicago, College of Medicine, Chicago, IL; 2 Department of Ophthalmology and Visual Sciences, University of Illinois at Chicago, College of Medicine, Chicago, IL

## Abstract

**Purpose:**

Cancer stem cells have increased resistance against a variety of anti-tumor treatment modalities. Vasculogenic mimicry (VM) patterns are present in numerous malignant tumor types, represent the formation of perfusion pathways by tumor cells, and their presence in tumors is associated with adverse outcome. Earlier we have shown that VM-forming tumor cells in three-dimensional (3D) uveal melanoma cultures have increased resistance against cytotoxic agents and oncolytic herpes simplex virus-mediated destruction. The purpose of the current study was to explore the possibility that this increased resistance of VM-forming tumor cells is due to a cancer stem cell phenotype.

**Methods:**

The expression of cancer stem cell marker cluster of differentiation 271 (CD271) was determined in traditional two-dimensional (2D) and 3D cultures of C918 uveal melanoma cells by fluorescent immunocytochemistry.

**Results:**

We found that the VM-forming tumor cell subpopulation in 3D cultures expressed CD271. In contrast, cells grown in 2D cultures and tumor cell subpopulations not participating in VM formation in 3D cultures were negative for CD271.

**Conclusions:**

These findings suggest that VM-forming uveal melanoma cells acquire a cancer stem cell-like phenotype that may play a role in the increased therapy resistance of these cells.

## Introduction

According to the cancer stem cell hypothesis, a subpopulation of malignant cells capable of self-renewal is responsible for tumor initiation, progression, and generation of phenotypic heterogeneity. Importantly, cancer stem cells also have increased resistance against a variety of anti-tumor treatment modalities [1,2]. As cancer stem cells represent a unique subpopulation of tumor cells, isolation and characterization of these cells has critical importance for the development of new therapeutic strategies [3].

One of the most studied tumor stem cell-markers is cluster of differentiation 271 (CD271). CD271 (known as also nerve growth factor receptor, NGFR or p75NTR) is a neurotrophin receptor, which can bind all of the neurotrophins by similar affinity [4]. It has contradictory actions; it functions to promote cell survival or induce cell death [5]. Expression of CD271 has been found in several human neural crest-derived tissues and in some human cancers, including melanomas [6]. Recently, CD271 has been used as an important cancer stem cell marker in melanoma [3,7,8].

Vasculogenic mimicry (VM) patterns are present in numerous malignant tumor types, represent the formation of perfusion pathways by tumor cells, and their presence in tumors is associated with adverse outcome [9-16]. While VM formation is clearly a marker of highly invasive tumor phenotype, mechanisms by which these structures may contribute to adverse outcome are not well understood. It has been proposed that VM formation may facilitate tumor perfusion and the physical connection between VM and blood vessels may also facilitate hematogeneous dissemination of tumor cells [14]. Recent studies suggest that malignant melanoma initiating cells (MMIC) are specifically associated with VM and it has been proposed that one mechanism by which MMIC promote tumor growth is by the induction of VM formation by MMIC [17]. Recent studies in our laboratory indicate that VM-forming tumor cells in three-dimensional (3D) uveal melanoma cultures have increased resistance against cytotoxic agents and oncolytic herpes simplex virus-mediated destruction [18,19]. These observations raise the possibility that the increased therapy resistance of VM-forming tumor cells is due to a cancer stem cell phenotype. To explore this possibility, the current study was designed to determine the expression of a known cancer stem cell marker, CD271 in traditional two-dimensional (2D) and 3D cultures of C918 uveal melanoma cells by fluorescent immunocytochemistry. C918 uveal melanoma cells have been reported to form VM in 3D cultures [18]. As controls, we also studied CD271 expression in another uveal melanoma cell line, OCM1 that does not form VM in culture [18]. We found that the VM-forming tumor cell subpopulation in 3D C918 cultures expressed CD271. In contrast, C918 cells in 2D cultures and tumor cell subpopulations not participating in VM formation in 3D cultures were negative for CD271. Neither 2D nor 3D cultures of OCM1 uveal melanoma cells expressed CD271. These findings suggest that a cancer stem cell-like phenotype may play a role in the increased therapy resistance of VM-forming tumor cells.

## Methods

### Cells

C918 and OCM1 uveal melanoma cell lines were maintained in Eagle's Minimal Essential Medium (BioWhittaker Inc., Walkersville, MD) supplemented with heat inactivated 15% fetal bovine serum (Fisher, Ontario, Canada) without the addition of exogenous ECM molecules or growth factors. These cell lines have been described in detail previously [20-23].

### 2D and 3D uveal melanoma cultures

Melanoma cells were grown on eight-well glass chamber slides (Lab-Tek II, Naperville, IL) in Eagle’s Minimal Essential Medium medium either in the presence (3D cultures) or in the absence (2D cultures) of ECM rich in laminin (Matrigel, BD Biosciences, Bedford, MA). For 3D cultures, Matrigel was poured onto tissue culture plates to a depth of approximately 0.2 mm followed by polymerization for 1 h at 37 °C before placement of melanoma cells on the Matrigel surface. Cultures were incubated in repeatedly refreshed culture medium three-times a week at 37 °C in a humidified atmosphere containing 5% CO_2_.

### Fluorescent immunocytochemistry

C918 and OCM1 uveal melanoma cells cultured with or without Matrigel were fixed in 2% formalin for 20 min in room temperature after forming VM structure under 3D culture conditions or confluent monolayer in 2D cultures. For CD271 labeling, cultures were incubated with monoclonal anti-human rat p75 NGF receptor as primary antibody (Abcam, Cambridge, MA) at 10 µg/ml final concentration for 1h at room temperature. For visualization of CD271 on uveal melanoma cells, cultures were incubated with FITC conjugated polyclonal anti-rat rabbit IgGs (Abcam) at 10 µg/ml final concentration for 1 h at room temperature in dark. Green fluorescence was detected by under an inverted fluorescent microscope (Leica, Bannockburn, IL).

## Results

### VM-forming C918 uveal melanoma cells express CD271 in 3D cultures

**Figure 1:**
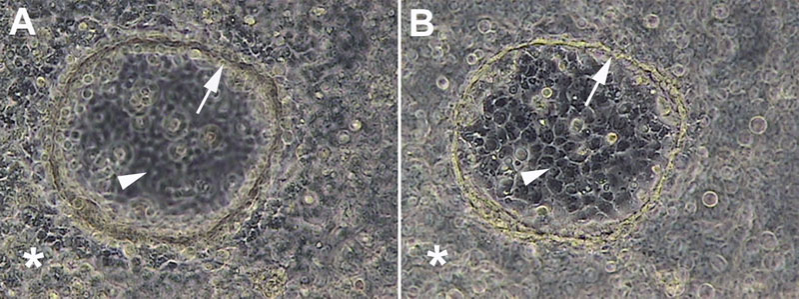
Morphology of 3D C918 uveal melanoma cultures. Pictures of a 3D C918 uveal melanoma culture with focus either on cells growing on the Matrigel surface (**A**) or on cells growing on the bottom of the tissue culture well (**B**). Vasculogenic mimicry (VM) patterns are marked by arrows, asterisks point to cells growing in monolayer on the Matrigel surface and arrowheads highlight cells growing on the bottom of the culture dish. Magnification: 200×.

**Figure 2:**
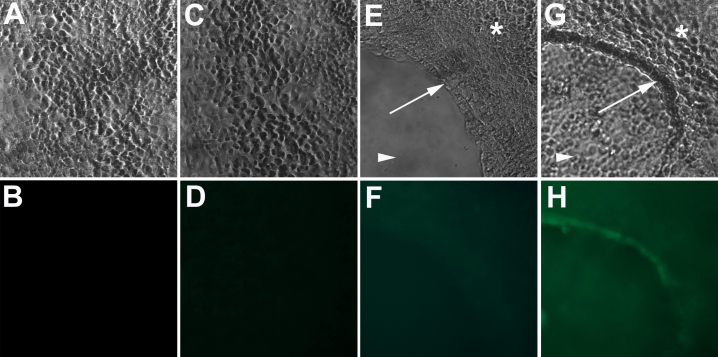
CD271 stem cell marker expression in C918 uveal melanoma cultures. The upper row of pictures demonstrates the morphology of two-dimensional (2D; **A** and **C**) and three-dimensional (3D; **E** and **G**) cultures of C918 uveal melanoma with the formation of prominent VM in 3D cultures. Arrows in **E** and **G** point to VM. Asterisks in **E** and **G** point to cells growing in monolayer on the Matrigel surface. Arrowheads in **E** and **G** point to cells growing on the bottom of the culture dish. Panel **H** demonstrates detection of CD271 expression by VM-forming tumor cells by immunofluorescence in 3D cultures. No CD271 expression was detected in 2D cultures (**D**). CD271 expression was also not detected in control 2D and 3D cultures where no primary antibody was used (**B** and **F**). Magnification: 200×.

To detect CD271 expression in C918 and OCM1 cultures, uveal melanoma cells were seeded onto plastic culture dishes to establish 2D cultures and onto Matrigel matrix surface to form 3D structures (Figure 1). Both C918 and OCM1 cell cultures became confluent in the plastic dish and formed monolayer. C918 melanoma cells cultured in the presence of Matrigel matrix formed a variety of morphologically distinguishable tumor cell subpopulations including VM (Figure 1A,B and Figure 2E,G). Specifically and as described in detail previously (Valyi-Nagy et al. [18]), these subpopulations included cells that grew in monolayer on the Matrigel surface, cells that formed VM on the Matrigel surface and tumor cells that grew as a monolayer on the bottom of the culture dish under the Matrigel layer (Figure 1A,B and Figure 2E,G). OCM1 cells in 3D cultures formed cell aggregates on the Matrigel surface and invaded Matrigel without forming VM. Both 2D and 3D cultures were labeled with CD271 antibody, then FITC-conjugated secondary antibody. For control cultures, no primary antibody was added. We found that C918 uveal melanoma cells cultured under 2D conditions did not express CD271 (Figure 2D). In contrast, a subpopulation of C918 cells cultured in 3D, specifically VM-forming cells expressed CD271 at clearly detectable level (Figure 2H). Interestingly, other subpopulations of uveal melanoma cells in 3D cultures, including monolayer forming cells on the Matrigel surface and on the bottom of the culture dish were CD271 negative (Figure 2H). Neither 2D nor 3D cultures of OCM1 uveal melanoma cells expressed CD271 (data not shown). Control 2D and 3D uveal melanoma cultures exposed only to secondary antibody remained CD271 negative (Figure 2B,F, respectively).

## Discussion

Here we show for the first time that a morphologically distinct subpopulation of uveal melanoma cells, VM-forming tumor cells express CD271, a known cancer stem cell marker in 3D cultures in the presence of laminin-rich ECM.

It is well known that tumor cells cultured in the presence of extracellular matrix components or on non-adherent surfaces form 3D structures. In these 3D cultures, tumor cells are more resistant to chemotherapeutic drugs and radiation, than in the traditionally used two-dimensional 2D cultures [24-27]. Interestingly, 3D spheroid cultures of tumor cells grown in stem cell media or in the presence of a hydrophobic substrate, polydimethylsiloxane can become enriched in cancer stem cells [3,28].

Recently we found that uveal melanoma cells grown in 3D cultures are more resistant to oncolytic effect of herpes simplex virus than cells grown in 2D cultures [18,23]. Furthermore, we have shown that VM-forming tumor cells in 3D uveal melanoma cultures have increased resistance against cytotoxic agents and oncolytic herpes simplex virus-mediated destruction [18,19]. These observations raise the possibility that there is a cause and effect connection between cancer stem cell enrichment in VM and increased resistance to chemotherapeutic agents and virotherapy in 3D melanoma cell cultures. To find the answer to this question, we aimed to determine whether the VM subpopulation of C918 cells with increased resistance to herpes simplex virus type 1 (HSV-1) have cancer stem cell-like phenotype. As controls, we also studied CD271 expression in another uveal melanoma cell line, OCM1 that does not form VM in culture [18].

Recent published work suggests that tumor stem cells are present in uveal melanoma [29,30]. In gene set enrichment analysis of human uveal melanoma samples, a stem cell-like phenotype was associated with metastasis [29]. In another study, another stem cell marker CD133 was expressed by some tumor cells in human uveal melanoma tissue indicating that uveal melanoma may contain cancer stem cells [30].

We used CD271 as cancer stem cell marker in the current work because the usefulness of CD271 as a melanoma cancer stem cell marker is well documented by recent reports [7,8]. Specifically, a CD271^+^ cell population isolated directly from aggressive melanoma samples and transplanted into T-, B- and natural-killer cell deficient mice resulted in melanoma formation at a markedly higher rate than CD271- cells [7]. Interestingly, most of CD271 expressing cells completely or partially lacked the expression of other melanoma tumor antigens, such as tyrosinase and melanoma antigen recognized by T cells (MART1) [7], indicating that CD271^+^ melanoma cells have a less differentiated phenotype. Important features of cancer stem cells include the ability to reproduce the full heterogeneity of the parental tumor and continuous growth even after multiple passages [8,31]. Using melanoma cell isolation and xenotransplantation, Civenni et al. [8] proved that CD271-positive melanoma cells meet the above described definition of the cancer stem cells [8].

In our experiments, C918 uveal melanoma cells did not express CD271 in traditional 2D cultures; however, in 3D cultures, VM-forming C918 uveal melanoma cells expressed CD271. OCM1 uveal melanoma cells did not form VM and did not express CD271. These data suggest that VM-forming C918 cells have a stem cell-like phenotype.

It is already known that VM-forming tumor cells from aggressive melanoma display increased levels of genes associated with undifferentiated embryonic-like phenotype [16]. Recent studies also indicated that malignant melanoma initiating cells (MMIC) are specifically associated with VM and it has been proposed that one mechanism by which MMIC promote tumor growth is by the induction of VM formation by MMIC [17].

In summary, our results indicate that uveal melanoma cells, under the 3D culture conditions that facilitate the generation of VM patterns, may express a phenotypic marker – CD271 – that is associated with a stem-cell phenotype. This observation is consistent with and extends previous reports suggesting a connection between cancer stem cells and VM. It is clear that our findings represent only initial observations and an extension of our studies to human uveal melanoma tissues as well as the use of additional stem cell-markers, such as CD133, CD166 and nestin will be needed to fully explore the association between VM-formation and cancer stem cell phenotype. However, even at this stage, our results raise the possibility that increased therapy-resistance of VM-forming tumor cells is due to a cancer stem cell-like phenotype.
